# Revisiting the diagnosis of ‘resistant hypertension’: what should we do nowadays’

**DOI:** 10.1038/s41371-021-00631-3

**Published:** 2021-11-06

**Authors:** Reem Alsharari, Eduard Shantsila, Gregory Y. H. Lip, Alena Shantsila

**Affiliations:** 1 Liverpool Centre for Cardiovascular Science, University of Liverpool, Liverpool Heart & Chest Hospital and Liverpool John Moores University, Liverpool, UK; 2 Institute of Cardiovascular Sciences, University of Birmingham, Birmingham, UK

**Keywords:** Health care, Diseases

Hypertension is one of the leading causes of death globally [1]. Despite the widespread use of antihypertensive drugs and treatment strategies, many patients with hypertension fail to achieve target blood pressure (BP) control, a condition called resistant hypertension (RH). RH is a major clinical problem as it is associated with a high risk of hypertension-mediated organ damage (HMOD), including chronic kidney disease (CKD), and premature cardiovascular diseases (CVD) [2, 3]. Therefore, early identification and effective management strategies have the potential to improve hypertension treatment and lower cardiovascular morbidity and mortality of the hypertensives population, especially RH.

Recent guidelines propose better strategies to define, detect, and manage RH (American College of Cardiology (ACC)/American Heart Association (AHA) [4, 5], International Society of Hypertension (ISH) [6], the European Society of Cardiology (ESC)/European Society of Hypertension (ESH) [7] and the National Institute for Health and Care Excellence (NICE) [8]).

## The definition and prevalence of resistant hypertension

**Fig. 1:**
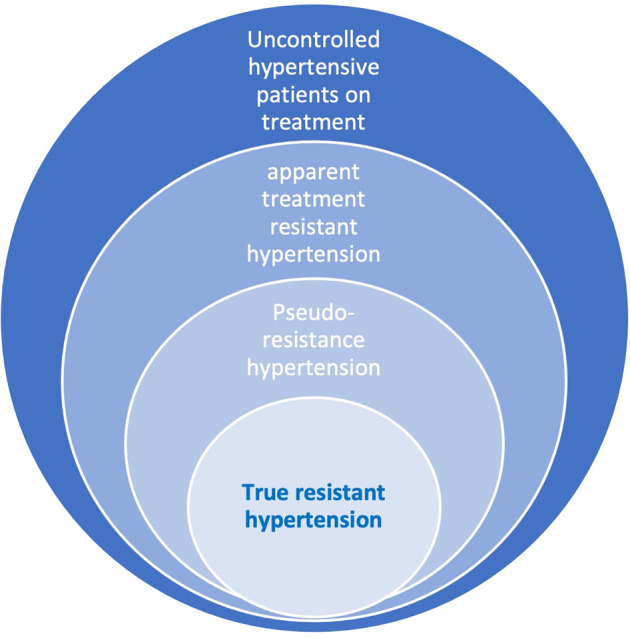
Types of uncontrolled hypertension. True resistant hypertension is estimated to occur in about 10% of the treated hypertension population.

The ESC/ESH and NICE guidelines [7, 8] keep the previous definition of hypertension and identify RH based on office systolic and diastolic BP exceeding 140/90 mmHg, respectively, despite the concurrent use of three or more different antihypertensive agents, one of which being a diuretic. The guidelines estimate the prevalence of true RH as about 10% of the treated hypertension (Fig. 1) [9].

Based on new evidence from large studies and meta-analyses such as Systolic Blood Pressure Intervention Trial [10], the AHA/ACC and ISH guidelines [5, 6] reduced BP thresholds diagnosis of hypertension (≥130/80 mmHg) and initiation of antihypertensive treatment (i.e. <130/80 mmHg). Treatment is recommended for high-risk patients, including older age (≥ 65 years), 10-year history of atherosclerotic CVD, ≥ 10% of calculated risk of atherosclerotic CVD, CKD, and diabetes mellitus. This updated definition modifies the diagnosis of RH and expands its prevalence. Following the AHA/ACC guidelines, RH is further classified based on the number of pharmaceutical agents prescribed into two subcategories: uncontrolled RH and controlled RH. Uncontrolled RH is defined as having an office BP above the goal in sitting position despite using a diuretic and two different antihypertensive medications at optimal doses. Controlled RH is diagnosed when it requires four or more antihypertensive medications to achieve optimal BP levels.

Based on the previous threshold of 140/90 mmHg and data taken from the National Health and Nutrition Examination Survey [11], the prevalence of RH in the United States was around 13%, and it is expected to increase by ~4% using the news definition. Therefore, RH definition may vary noticeably between studies and the this needs to be considered. AHA/ACC also introduced the apparent treatment RH (aTRH), which is used when there is a potential pseudo-resistance, and RH diagnosis cannot be excluded due to missing one or more of the diagnostics criteria.

## True resistant hypertension and pseudo resistant hypertension

A study by Bhatt et al. [12] estimated that pseudo-resistance hypertension prevalence is almost 33% among patients diagnosed with RH. Another study by Sierra et al. [13] based on a database from the Spanish ambulatory BP monitoring (ABPM) Registry among more than 8000 patients diagnosed with RH (based on office BP), only 63% had true RH and 37% were pseudo-resistance following ABPM. In 2018, a meta-analysis [14] included around 3.2 million patients with treated hypertension who participated in 91 studies between 1991 and 2017 reported the overall prevalence of true RH was 10.3%. The prevalence of true RH was higher in patients with CKD (22.9%), renal transplant (56.0%), and elderly individuals (12.3%).

**Fig. 2:**
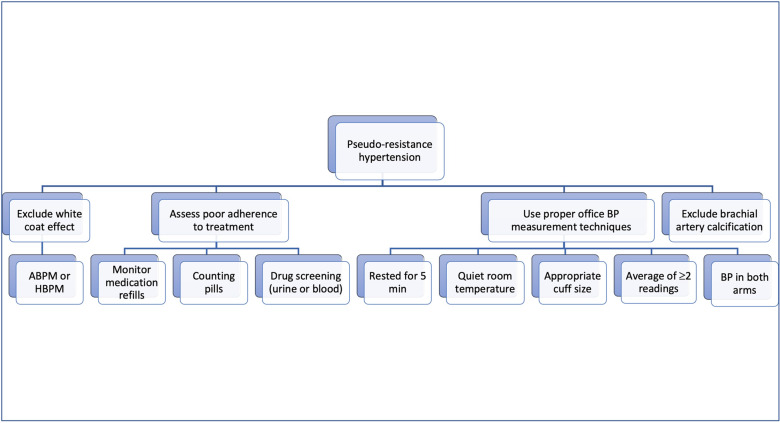
Detection of pseudo-resistance hypertension in patients on drug therapy. ABPM: ambulatory blood pressure monitoring, BP: blood pressure, HBPM: home blood pressure monitoring.

The study showed a high prevalence of TRH and pseudo-RH, ~14.7% and 10.3%, respectively [14]. This indicates the significance of excluding pseudo-RH. Pseudo-RH can be caused by many factors. This includes inadequate office BP measurement techniques, white coat hypertension or inappropriate combinations of antihypertensive medications (Fig. 2).

## What else to consider when resistant hypertension is suspected

To evaluate the BP measurement technique, Boonyasai et al. [15] observed six primary care clinics over for 6 months. The percentage of the staff adherence to the correct protocol was around 70%. To confirm the diagnosis of RH, it is essential to follow accurate BP measurement techniques. All guidelines agreed that patients should be rested for at least 5 min before taking BP measurements in a quiet and adequate room temperature. BP measurements should include measuring BP in both upper arms using appropriate cuff size (too small or too large cuff can lead to incorrect increased BP values). The AHA/ACC recommends that an average of two or more readings should be measured on ≥2 separate visits. The ESC/ESH recommended taking three BP readings 1–2 min apart. Additional readings are recommended if there is >10 mmHg difference between the first two readings. The white coat effect is present in 28–39% of patients with RH [13, 16]. It is important to consider a possibility of white coat effect and to use out-of-office BP monitoring (with ambulatory or home BP monitoring) to confirm the true RH.

Treatment nonadherence needs to be considered. A retrospective analysis conducted in United Kingdom and Czech Republic showed lack of adherence to antihypertensive treatments in ~50% of patients [17]. The risk of treatment nonadherence is particularly high with complex treatment schemes due to high pill burden and adverse drugs reactions, when there is suboptimal patient-physician communication or lack of patients’ understanding of the treatment. Several methods to assist management of suspected poor pharmacological adherence, including monitoring medication refills, counting pills, and drug screening of antihypertensive agents (urine or blood samples). Finally, brachial artery calcification may cause incorrect elevated readings during office BP measurements, especially in older patients [18].

## Management of resistant hypertension

**Fig. 3:**
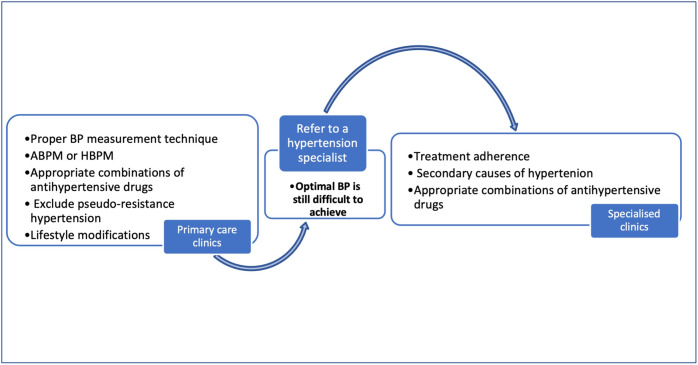
Management pathways of patients with resistant hypertension. ABPM: ambulatory blood pressure monitoring, BP: blood pressure, HBPM: home blood pressure monitoring.

The guidelines recommend that if it is difficult to achieve a BP target despite using three antihypertensive agents the patient should be referred to a hypertension specialist for further investigation and management (Fig. 3). This should parallel assessments for factors that can affected BP control. It is important to assess lifestyle factors that might be contributing to RH. Nutritional approaches to reduce BP include limiting alcohol consumption, consuming more potassium and less sodium. It is also important to reduce stress levels, maintain healthy weight and physically active lifestyle.

Inappropriate combinations of antihypertensive drugs are common in older patients, especially in those with polypharmacy. An observational study by Marquez et al. [19] identified potentially inappropriate drug combinations in 51.3% of older patients with over-prescription reported in 27.8% and under-prescription in 35%. Overuse of nonsteroidal anti-inflammatory (NSAIDs) medications can lead to increased BP and interfere with antihypertensive agents [20].

Secondary causes of hypertension include renal artery stenosis, renal parenchymal disease, primary hyperaldosteronism, Cushing’s syndrome, hypothyroidism, hyperthyroidism, coarctation of aorta and obstructive sleep apnoea. The guidelines recommended to evaluate HMOD such as measuring renal function, albuminuria, presence of retinopathy, carotid intima–media thickening and left ventricular hypertrophy.

For pharmacological treatments include combinations of at least three different agents (a long-acting thiazide diuretics, long-acting calcium channel blocker, and a renin-angiotensin system blocker with further addition of a low dose of mineralocorticoid receptor antagonist (spironolactone). If spironolactone was not tolerated other agents should be considered, usually as part of a specialist care. Device-based interventions, such as renal denervation, carotid baroreceptors stimulators, and central arteriovenous fistula are still under development.

In conclusion, RH remains a common and clinically significant problem. It should be addressed by careful consideration of the multitude of associated factors. Unfortunately, management of true RH is still challenging, and further research is essential to understanding its pathophysiology and targeted treatments.

## References

[1] Roth GA, Mensah GA, Johnson CO, Addolorato G, Ammirati E, Baddour LM (2020). Global Burden of Cardiovascular Diseases and Risk Factors, 1990-2019: update From the GBD 2019 Study. J Am Coll Cardiol.

[2] Muiesan ML, Salvetti M, Rizzoni D, Paini A, Agabiti-Rosei C, Aggiusti C (2013). Resistant hypertension and target organ damage. Hypertens Res.

[3] Daugherty SL, Powers JD, Magid DJ, Tavel HM, Masoudi FA, Margolis KL (2012). Incidence and prognosis of resistant hypertension in hypertensive patients. Circulation.

[4] Whelton PK, Carey RM, Aronow WS, Casey DE, Collins KJ, Dennison Himmelfarb C (2018). 2017 ACC/AHA/AAPA/ABC/ACPM/AGS/APhA/ASH/ASPC/NMA/PCNA Guideline for the Prevention, Detection, Evaluation, and Management of High Blood Pressure in Adults: Executive Summary: a Report of the American College of Cardiology/American Heart Association Task Force on Clinical Practice Guidelines. Circulation.

[5] Carey RM, Calhoun DA, Bakris GL, Brook RD, Daugherty SL, Dennison-Himmelfarb CR (2018). Resistant Hypertension: Detection, Evaluation, and Management: a Scientific Statement From the American Heart Association. Hypertension.

[6] Unger T, Borghi C, Charchar F, Khan NA, Poulter NR, Prabhakaran D (2020). 2020 International Society of Hypertension global hypertension practice guidelines. J Hypertens.

[7] Williams B, Mancia G, Spiering W, Agabiti Rosei E, Azizi M, Burnier M (2018). 2018 ESC/ESH Guidelines for the management of arterial hypertension. Eur Heart J.

[8] Hypertension in adults: diagnosis and management. National Institute for Health and Care Excellence: Clinical Guidelines. London, 2019. Avilable at: https://www.nice.org.uk/guidance/ng136.

[9] Achelrod D, Wenzel U, Frey S (2015). Systematic review and meta-analysis of the prevalence of resistant hypertension in treated hypertensive populations. Am J Hypertens.

[10] Wright JT, Whelton PK, Reboussin DM (2016). A Randomized Trial of Intensive versus Standard Blood-Pressure Control. N Engl J Med.

[11] Persell SD (2011). Prevalence of resistant hypertension in the United States, 2003-2008. Hypertension.

[12] Bhatt H, Siddiqui M, Judd E, Oparil S, Calhoun D (2016). Prevalence of pseudoresistant hypertension due to inaccurate blood pressure measurement. J Am Soc Hypertens.

[13] de la Sierra A, Segura J, Banegas JR, Gorostidi M, de la Cruz JJ, Armario P (2011). Clinical features of 8295 patients with resistant hypertension classified on the basis of ambulatory blood pressure monitoring. Hypertension.

[14] Noubiap JJ, Nansseu JR, Nyaga UF, Sime PS, Francis I, Bigna JJ (2019). Global prevalence of resistant hypertension: a meta-analysis of data from 3.2 million patients. Heart.

[15] Boonyasai RT, Carson KA, Marsteller JA, Dietz KB, Noronha GJ, Hsu YJ (2018). A bundled quality improvement program to standardize clinical blood pressure measurement in primary care. J Clin Hypertens (Greenwich).

[16] Salles GF, Cardoso CR, Muxfeldt ES (2008). Prognostic influence of office and ambulatory blood pressures in resistant hypertension. Arch Intern Med.

[17] Gupta P, Patel P, Strauch B, Lai FY, Akbarov A, Gulsin GS (2017). Biochemical Screening for Nonadherence Is Associated With Blood Pressure Reduction and Improvement in Adherence. Hypertension.

[18] Messerli FH, Ventura HO, Amodeo C (1985). Osler’s maneuver and pseudohypertension. N Engl J Med.

[19] Marquez PHP, Torres OH, San-Jose A, Vidal X, Agusti A, Formiga F (2017). Potentially Inappropriate Antihypertensive Prescriptions to Elderly Patients: Results of a Prospective, Observational Study. Drugs Aging.

[20] Forman JP, Stampfer MJ, Curhan GC (2005). Non-narcotic analgesic dose and risk of incident hypertension in US women. Hypertension.

